# Methodological Quality of Heart Failure Systematic Reviews and Meta-Analyses in the Era of AMSTAR-2 (a Measurement Tool to Assess Systematic Reviews): A Pre-registered Repeat Cross-Sectional Survey

**DOI:** 10.7759/cureus.117099

**Published:** 2026-09-28

**Authors:** Md Shadman Shakib, Raidah Sadia

**Affiliations:** 1 General Practice, Sheffield Teaching Hospitals NHS Foundation Trust, Sheffield, GBR; 2 Acute Medicine, Sheffield Teaching Hospitals NHS Foundation Trust, Sheffield, GBR

**Keywords:** amstar-2, heart failure, meta-analysis, meta-research, methodological quality, systematic review

## Abstract

Background

Systematic reviews and meta-analyses guide heart failure (HF) practice, yet their methodological quality has been questioned. An AMSTAR-2 (A Measurement Tool to Assess Systematic Reviews) appraisal of HF reviews in 11 high-impact journals during 2009-2019 rated 79 of 81 as critically low confidence. Whether quality has changed in the era of routine protocol registration and PRISMA 2020 is unknown.

Methods

In a pre-registered repeat cross-sectional survey, we replicated that sampling frame for 2021-2025: HF systematic reviews with a meta-analysis of an intervention, in the same 11 journals. Two reviewers independently applied all 16 AMSTAR-2 items to each review; disagreements were resolved by consensus. Item-level compliance (the proportion rated Yes or Partial Yes) and overall confidence were compared with the 2009-2019 figures.

Results

Of 751 records screened, 26 reviews were included (median of eight primary studies). Overall confidence was critically low in 16, low in seven, and moderate in three; none was high. A total of 10 of 26 (38%) reached low confidence or better, versus two of 81 (2%) in 2009-2019. Compliance was higher for an a priori protocol (5% to 88%), the provision of an excluded-studies list (5% to 46%), and the specification of population, intervention, comparison, and outcome (PICO) (65% to 100%). Conversely, accounting for the risk of bias when interpreting results was lower (88% to 35%). Reporting the funding of individual included studies remained rare in both eras (4%). Inter-rater agreement was moderate (kappa = 0.47).

Conclusions

The AMSTAR-2 compliance of high-impact HF systematic reviews was higher in 2021-2025 than in 2009-2019, most markedly for protocol reporting, though most reviews remained critically low. Reviews were less likely to carry risk-of-bias findings into their interpretation; one interpretation is that registration has become routine while substantive appraisal has not.

## Introduction

Systematic reviews and meta-analyses sit at the top of the evidence hierarchy and directly inform heart failure (HF) guidelines and clinical practice. Their influence makes their methodological quality consequential: a review that is poorly conducted or incompletely reported can propagate biased estimates into clinical decision-making. The AMSTAR-2 instrument (A Measurement Tool to Assess Systematic Reviews) was developed to appraise the conduct of systematic reviews of interventions across 16 domains, seven of which are designated critical, and it yields an overall rating of high, moderate, low, or critically low confidence [1].

A 2022 appraisal applied AMSTAR-2 to 81 HF systematic reviews with meta-analysis published in 11 high-impact journals between January 2009 and July 2019 and rated 79 as critically low confidence and two as low [2]. That window, however, closed less than two years after AMSTAR-2 was published in 2017 and before the release of PRISMA 2020, and it predates the now-widespread adoption of prospective protocol registration [3]. Whether the methodological quality of HF reviews has changed in the intervening period is unknown. Appraisal tools and reporting guidance are widely assumed to raise standards, yet this assumption is seldom tested against a comparable earlier sample.

We therefore conducted a pre-registered repeat cross-sectional survey that replicated the earlier sampling frame for the period 2021-2025, comparing item-level AMSTAR-2 compliance and overall confidence between the two eras.

## Materials and methods

Study design and registration

This was a repeat cross-sectional methodological survey (meta-research); the unit of analysis is the published systematic review, not the patient. The protocol was registered on the Open Science Framework (OSF) on 4 September 2026, after the literature search and screening were complete and before any AMSTAR-2 rating began; the appraisal method, the comparison, and the analysis plan were fixed in advance. Deviations from the registered plan identified during peer review are documented in a dated update to the registration and are stated where relevant below. The survey followed the sampling and appraisal approach of the 2009-2019 study it replicates, with three intentional differences disclosed in the Limitations [2].

Sampling frame and search

We searched PubMed on 4 September 2026, restricted to the same 11 journals appraised in the earlier study: *Annals of Internal Medicine*, *Circulation*,* Circulation: Heart Failure*, the *European Heart Journal*, the *European Journal of Heart Failure*, the *Journal of the American College of Cardiology*, *JACC: Heart Failure*, *JAMA*, *JAMA Internal Medicine*, the *Lancet*, and the *New England Journal of Medicine*. We sought HF systematic reviews with meta-analysis published between 1 January 2021 and 31 December 2025, including records first available online within this window but carrying a 2026 print date. Retrieval yielded 751 records after de-duplication. As a check on search sensitivity, all 106 records tagged as meta-analyses by the PubMed publication-type filter were confirmed to have been retrieved; the tag was not used as the sampling frame because it missed 23 records whose titles declared a meta-analysis.

Title and abstract screening was supported by an automated first-pass tool that proposed an eligibility decision and reason for each of the 751 records; every proposed decision was reviewed and confirmed or overridden by an investigator, and the evidence basis of each final decision (full text, abstract, bibliographic record, or title only) was recorded. This automated support was confined to screening and is distinct from the independent human AMSTAR-2 appraisal described below; no automated tool contributed to any item-level rating.

Eligibility criteria

We included full journal articles that were systematic reviews reporting a quantitative synthesis (meta-analysis) of an intervention in a population of patients with heart failure. We excluded reviews with no meta-analysis; reports that were not systematic reviews (no search strategy or primary trial reports, post hoc analyses, cohort studies, editorials, correspondence, or corrections); prognostic, diagnostic, etiological, or descriptive questions; individual participant data meta-analyses; network meta-analyses; and umbrella reviews or overviews of existing meta-analyses. Eligibility otherwise matched the earlier study, which likewise excluded individual participant data and network meta-analyses. The one narrowing was a population-based definition of heart failure: the population of the included primary studies had to be patients with heart failure, rather than heart failure appearing among outcomes reported in a population defined by another condition. This definition was chosen because heart failure endpoints appear in most contemporary cardiometabolic trials, so an outcome-inclusive definition could not be applied reproducibly at screening; its potential influence on comparability is discussed in the Limitations.

Appraisal

Two reviewers (MSS and RS) independently applied all 16 AMSTAR-2 items to each included review and its supplementary material, without sight of one another's ratings; the second reviewer worked from a blinded form containing only the reviews, the items, and their permitted response options. Each reviewer's independent ratings were checked against the valid response set for each item; invalid responses (a Partial Yes on a binary-response item) occurred during independent rating in a minority of cells and were resolved during consensus reconciliation by the two reviewers jointly rereading the item against the source. Disagreements were resolved by discussion; a third arbiter was available but was not required. The original independent ratings are preserved unaltered in the deposited dataset.

Overall confidence was assigned using the AMSTAR-2 decision rule based on the seven critical domains (items 2, 4, 7, 9, 11, 13, and 15) [1]: high, no or one non-critical weakness and no critical flaw; moderate, more than one non-critical weakness but no critical flaw; low, one critical flaw with or without non-critical weaknesses; and critically low, more than one critical flaw. A critical flaw was a rating of "No" on a critical item. A "Partial Yes" on any item was pre-specified to count as a non-critical weakness rather than a critical flaw; a sensitivity analysis recomputed overall confidence under the stricter alternative.

Items 12 and 13 were scored "Yes" via the AMSTAR-2 low-risk-only route only where the review explicitly connected a risk-of-bias finding to the interpretation of a specific result, for example, through a certainty rating, a risk-of-bias-motivated sensitivity analysis, or the discussion of a specific study-level limitation; a standalone statement that all included studies were low risk, appearing once and not revisited when interpreting results, was not considered sufficient. This rule was applied uniformly to all reviews.

Statistical analysis

The primary outcome was the proportion of reviews rated Yes or Partial Yes on each item, matching the combined basis reported by the 2009-2019 study, compared with its published figures [2]. Proportions are reported with 95% Wilson score confidence intervals (CI); between-era differences are reported in percentage points with 95% Newcombe hybrid-score intervals, treating the two eras as independent samples, with comparator intervals derived from the published proportions and sample size. These intervals quantify sampling uncertainty within each restricted 11-journal sample only; they do not account for differences in retrieval method, population definition, or appraisal team between eras, and no inferential hypothesis tests were performed. Inter-rater agreement before consensus was summarized as raw agreement and Cohen's kappa, computed per item and as a single pooled estimate across all item-level ratings, interpreted using the Landis and Koch bands [4].

Three sensitivity analyses were pre-specified: including reviews excluded solely because heart failure was an outcome rather than the population, counting a Partial Yes in a critical domain as a critical flaw, and excluding the single most-represented journal. As an exploratory, non-pre-specified addition, whether each review reported a registry identifier in its methods was recorded separately from its item 2 rating. Analyses were performed in Python version 3.11 (Python Software Foundation, Fredericksburg, VA) using the pandas and NumPy libraries; the dataset and analysis code are openly available on OSF.

## Results

Of 751 records screened, 28 reviews initially met eligibility (Figure 1). On re-examination during peer review, two were found not to meet the pre-registered criteria and were excluded: One synthesized trials across a mixed population of type 2 diabetes, chronic kidney disease, and heart failure, and one was an umbrella review re-pooling existing meta-analyses. The primary analysis therefore comprises 26 reviews [5-30]. The included reviews synthesized a median of eight primary studies (range: 3-92). Sixteen were published in the *European Journal of Heart Failure*, four in the *European Heart Journal*, two in *JACC: Heart Failure*, and one each in *Annals of Internal Medicine*, *Circulation: Heart Failure*, the *Journal of the American College of Cardiology*, and the *Lancet*. No eligible reviews were retrieved from *JAMA*, *JAMA Internal Medicine*, or the *New England Journal of Medicine*.

**Figure 1:**
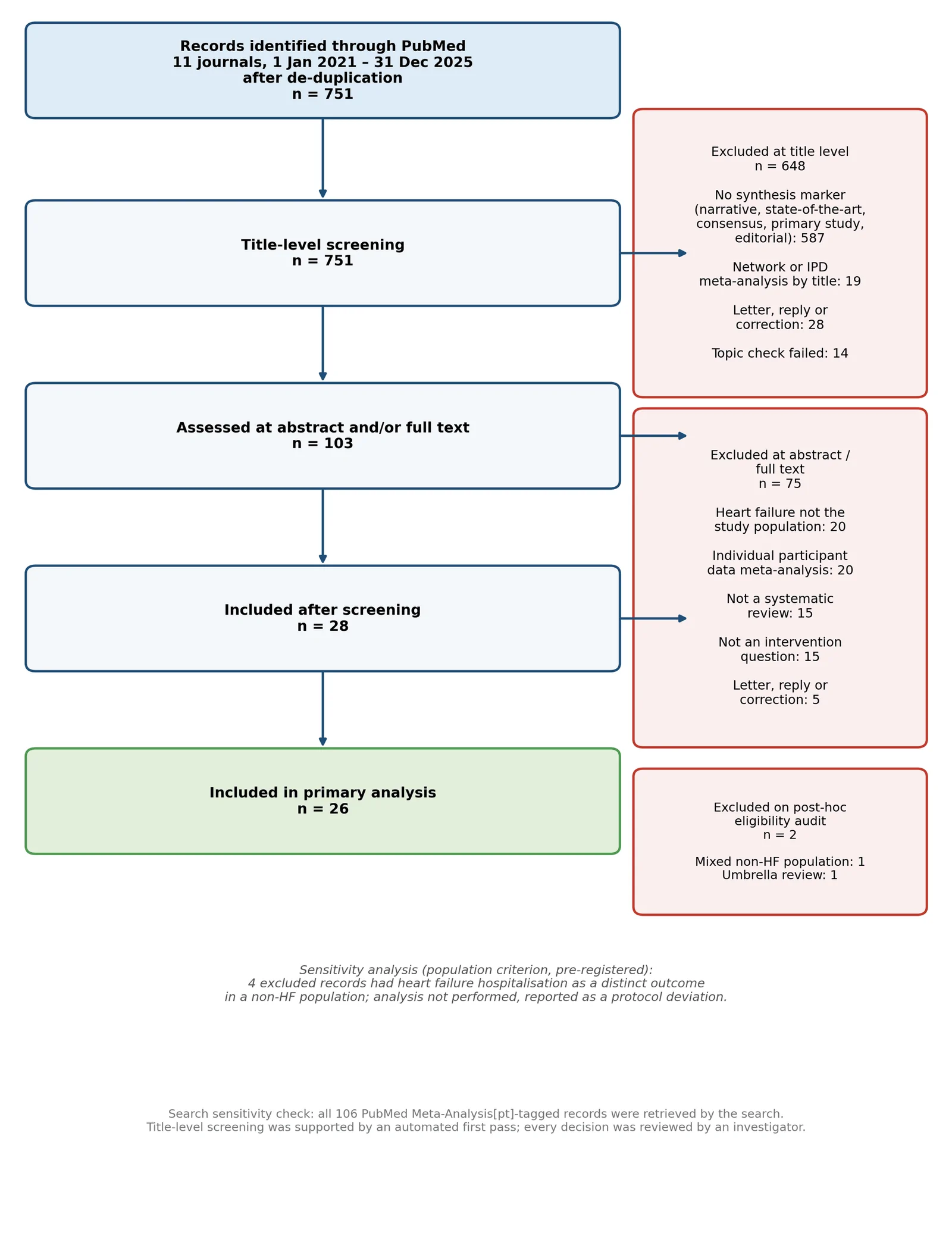
PRISMA 2020 flow diagram. Of 751 records identified, 648 were excluded at title screening, 75 at abstract or full-text assessment, and two in the post-hoc eligibility audit, leaving 26 reviews in the primary analysis. Of the records excluded because heart failure was not the study population, four reported heart failure as a distinct outcome and therefore met the eligibility criterion for the pre-specified population sensitivity analysis; that analysis was not performed and is reported as a protocol deviation.

Overall confidence

Overall confidence was critically low in 16 reviews (61.5%; 95% CI: 42.5 to 77.6), low in seven (26.9%; 95% CI: 13.7 to 46.1), and moderate in three (11.5%; 95% CI: 4.0 to 29.0); none reached high confidence. Ten of 26 reviews (38.5%; 95% CI: 22.4 to 57.5) achieved low confidence or better, compared with two of 81 (2.5%) in 2009-2019, a difference of +36.0 percentage points (95% CI: +18.8 to +55.1) (Figure 2).

**Figure 2:**
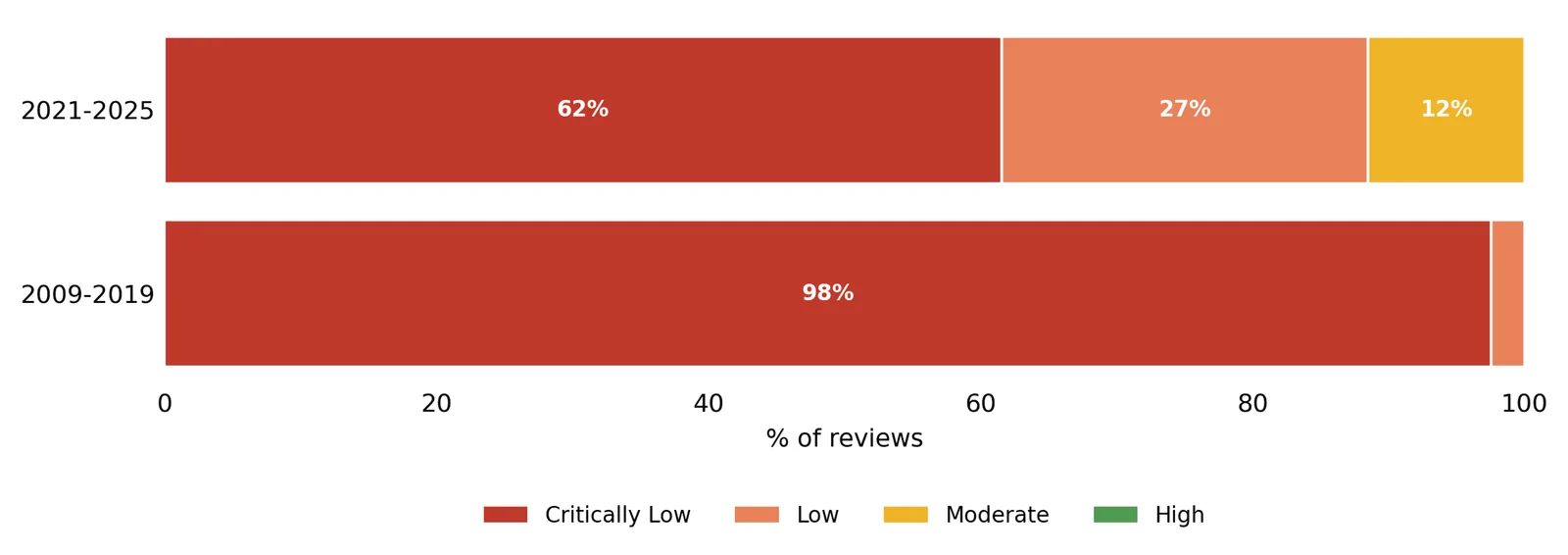
Overall AMSTAR-2 confidence rating: 2021-2025 (this survey, n = 26) versus 2009-2019. 2009-2019 data reproduced from Li et al., published under a Creative Commons Attribution 4.0 International (CC BY 4.0) license [2].

Item-level compliance

Item-level compliance, defined as the proportion of reviews rated Yes or Partial Yes, is shown against the 2009-2019 figures in Table 1 and Figure 3. The largest differences, by percentage points, were in a priori protocol reporting (+83.5; 95% CI: +64.7 to +91.6), the provision of an excluded-studies list (+41.2; 95% CI: +22.4 to +59.8), the specification of population, intervention, comparison, and outcome (PICO) components (+34.6; 95% CI: +18.6 to +45.4), and the justification of study design (+31.3; 95% CI: +11.6 to +50.7). In the opposite direction, accounting for risk of bias when interpreting results was lower by 53.0 percentage points (95% CI: -69.2 to -31.9). Reporting the funding of individual included studies was rare in both eras (15% and 4%; difference, -11.0; 95% CI, -20.8 to +5.3). Several smaller apparent differences, comprehensive search, duplicate selection, duplicate extraction, heterogeneity discussion, publication bias, and conflict-of-interest reporting, had confidence intervals spanning zero and are not distinguishable from sampling variation at this sample size.

**Table 1:** AMSTAR-2 item-level compliance (percentage rated Yes or Partial Yes): 2021-2025 (n = 26) versus 2009-2019 (n = 81). *Critical domains. PICO, population, intervention, comparison, and outcome; CI, confidence interval; RoB, risk of bias

AMSTAR-2 item	2021-2025, % (95% CI)	2009-2019, % (95% CI)	Difference, pp (95% CI)
1. PICO components	100.0 (87.1 to 100.0)	65 (54.6 to 74.9)	+34.6 (+18.6 to +45.4)
2. Protocol a priori*	88.5 (71.0 to 96.0)	5 (1.9 to 12.0)	+83.5 (+64.7 to +91.6)
3. Design justified	46.2 (28.8 to 64.5)	15 (8.7 to 24.1)	+31.3 (+11.6 to +50.7)
4. Comprehensive search*	92.3 (75.9 to 97.9)	86 (77.3 to 92.2)	+5.9 (-11.6 to +16.6)
5. Duplicate selection	76.9 (57.9 to 89.0)	68 (57.1 to 77.1)	+9.0 (-12.0 to +25.2)
6. Duplicate extraction	84.6 (66.5 to 93.9)	73 (62.3 to 81.3)	+11.8 (-8.3 to +25.8)
7. Excluded studies listed*	46.2 (28.8 to 64.5)	5 (1.9 to 12.0)	+41.2 (+22.4 to +59.8)
8. Studies described	100.0 (87.1 to 100.0)	83 (73.1 to 89.4)	+17.3 (+2.8 to +26.9)
9. RoB technique*	92.3 (75.9 to 97.9)	69 (58.4 to 78.1)	+23.2 (+4.4 to +35.3)
10. Funding of studies	3.8 (0.7 to 18.9)	15 (8.7 to 24.1)	-11.0 (-20.8 to +5.3)
11. MA methods*	96.2 (81.1 to 99.3)	78 (67.6 to 85.5)	+18.4 (+1.5 to +29.0)
12. RoB impact	15.4 (6.1 to 33.5)	27 (18.7 to 37.7)	-11.8 (-25.8 to +8.3)
13. RoB in interpretation*	34.6 (19.4 to 53.8)	88 (78.7 to 93.2)	-53.0 (-69.2 to -31.9)
14. Heterogeneity	96.2 (81.1 to 99.3)	85 (75.9 to 91.3)	+11.0 (-5.3 to +20.8)
15. Publication bias*	53.8 (35.5 to 71.2)	60 (49.6 to 70.4)	-6.6 (-27.5 to +13.9)
16. Conflict of interest	100.0 (87.1 to 100.0)	99 (93.3 to 99.8)	+1.2 (-11.7 to +6.7)

**Figure 3:**
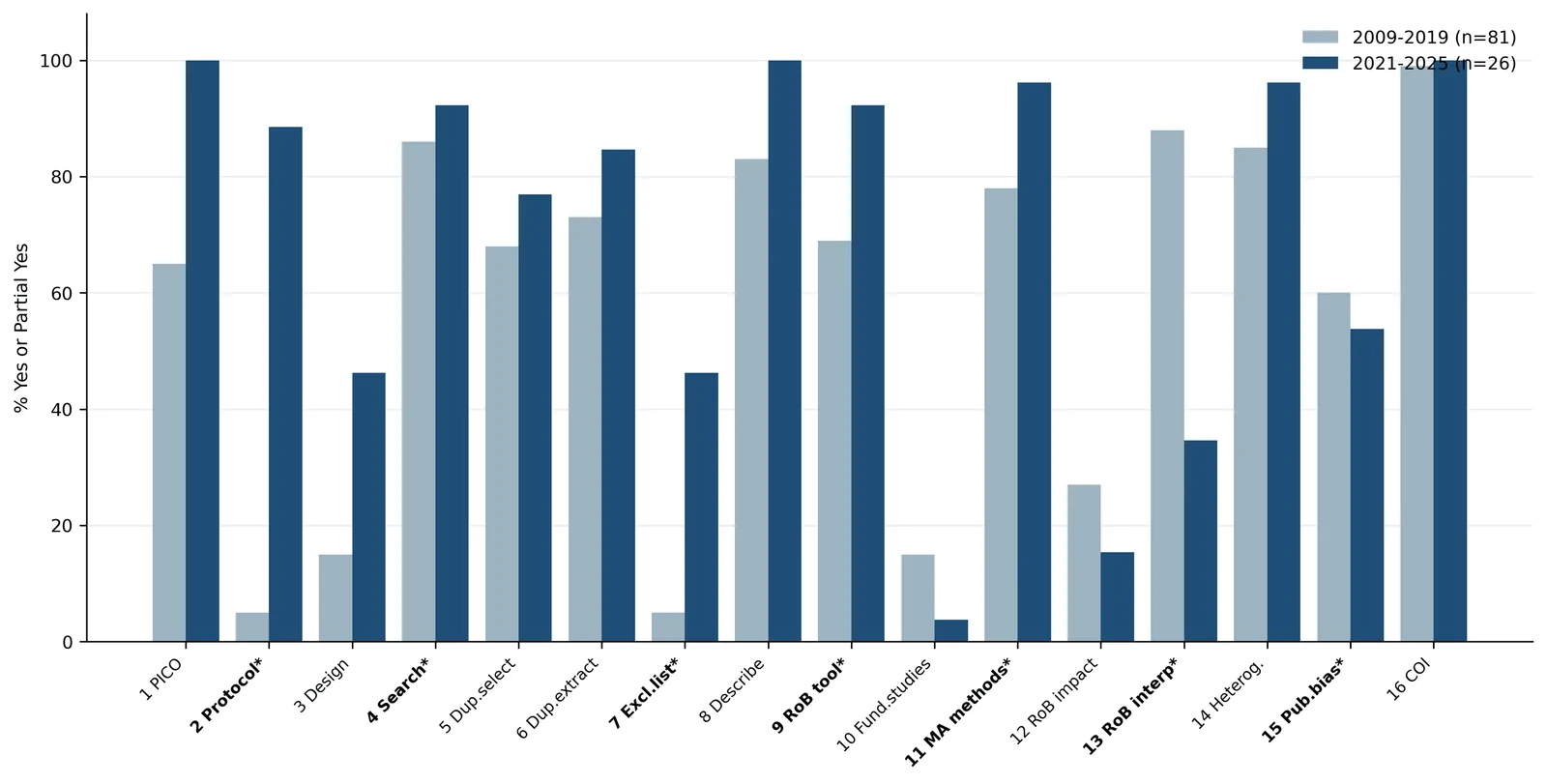
AMSTAR-2 item-level compliance (percentage rated Yes or Partial Yes): 2021-2025 (this survey, n = 26) versus 2009-2019 (n = 81). 2009-2019 data reproduced from Li et al., published under a Creative Commons Attribution 4.0 International (CC BY 4.0) license [2]. *Critical domains. PICO, population, intervention, comparison, and outcome; RoB, risk of bias; COI, conflict of interest

Protocol registration

As an exploratory, non-pre-specified check, 19 of 26 reviews (73.1%; 95% CI: 53.9 to 86.3) reported an International Prospective Register of Systematic Reviews (PROSPERO) identifier in their methods. This is lower than the combined item 2 compliance of 88.5%, reflecting that item 2 also credits reviews describing methods established a priori without formal registration; the two figures are distinct, and the former should not be read as equivalent to the latter.

Inter-rater agreement

Raw agreement between reviewers before consensus was 71.9% across 416 item-level ratings, and the pooled Cohen's kappa was 0.47 (moderate). This pooled estimate includes the four items on which one or both reviewers used a single category throughout; excluding them entirely gives a comparable value of 0.44. Agreement was highest for procedural items and lowest for judgment-dependent domains: the justification of design, excluded-studies list, risk of bias in interpretation, and publication bias. Item 13 (risk of bias in interpretation), the domain underlying the paper's principal negative finding, had the lowest pre-consensus agreement (57.7%; kappa: 0.18) and required extended discussion to reach consensus on 11 of 26 reviews. For items 1, 5, 8, and 11, one or both reviewers used a single category across all reviews; kappa is undefined for item 1 (both raters constant) and degenerate at zero for items 5, 8, and 11 (one rater constant), and raw agreement (100%, 73%, 88%, and 88%, respectively) is the meaningful statistic (Figure 4).

**Figure 4:**
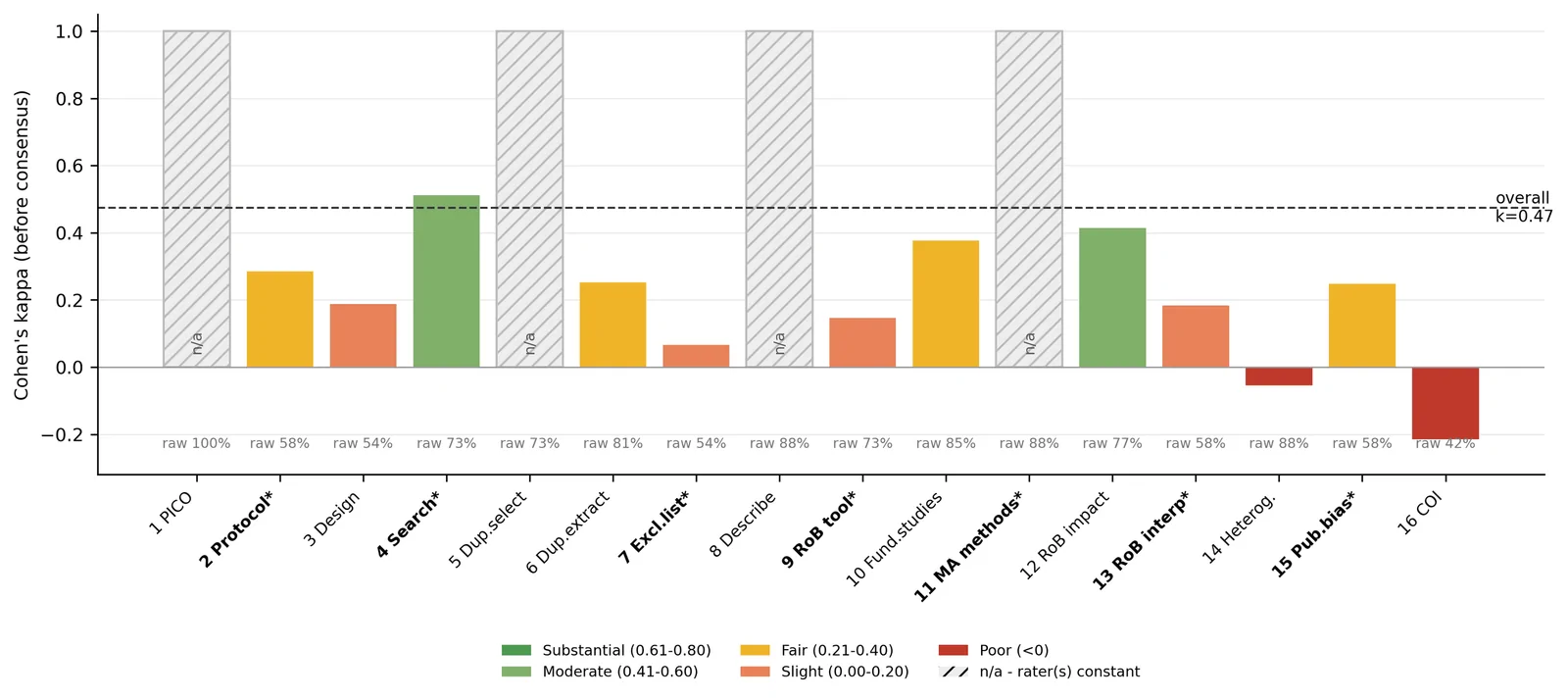
Inter-rater agreement (Cohen's kappa) by AMSTAR-2 item, this survey, before consensus. Items where a rater used one category throughout are flagged n/a; raw agreement is shown beneath each bar. Bands per Landis and Koch [4]. PICO, population, intervention, comparison, and outcome; RoB, risk of bias; COI, conflict of interest; n/a, not available

Sensitivity analyses

Under the stricter rule of counting a Partial Yes in a critical domain as a critical flaw, 25 of 26 reviews were critically low and one low. Excluding the *European Journal of Heart Failure*, which contributed 16 of 26 reviews, preserved the direction of every key finding among the remaining 10 reviews: A priori protocol compliance was 90%, the provision of an excluded-studies list was 50%, and accounting for risk of bias in interpretation was 20%, against 2009-2019 figures of 5%, 5%, and 88%, respectively.

The pre-registered population-criterion sensitivity analysis was designed to test whether relaxing the population definition to admit reviews in which heart failure was a reported outcome, rather than the study population, altered the findings. Four screened records met this broadened criterion: reviews in type 2 diabetes, acute coronary syndrome, and dialysis-dependent kidney failure populations that reported heart failure hospitalization as a distinct outcome. Because these populations differ substantively from the heart failure population the survey was designed to characterize, and because the primary finding was robust to the two other sensitivity analyses, this analysis was not performed. We report this as a deviation from the registered analysis plan, documented in a dated update to the registration.

## Discussion

In this pre-registered partial replication, AMSTAR-2 compliance among high-impact heart failure systematic reviews was higher in 2021-2025 than in the 2009-2019 sample appraised by Li et al. [2]. While the earlier appraisal found only two of 81 reviews above critically low confidence, 10 of 26 (38%) in the contemporary sample reached low confidence or better. The largest single difference was in a priori protocol reporting, from 5% to 88%. This coincides with the mainstreaming of prospective registration through PROSPERO and the expectation, now embedded in many journal policies, that reviews be registered before they begin; we note the coincidence without inferring causation, since the design cannot attribute the difference to registration practice, reporting guidance, or any other factor. Differences in the provision of an excluded-studies list, the specification of PICO components, and the justification of study design point in the same direction.

The most notable finding runs in the opposite direction. Compliance with accounting for risk of bias when interpreting results was 88% in 2009-2019 and 35% in 2021-2025, a difference whose confidence interval (-69 to -32 percentage points) lies well clear of zero. Contemporary reviews were more likely to register a protocol and less likely to carry the results of their own risk-of-bias assessment into their conclusions. One plausible interpretation is that the growth of registration and reporting checklists has concentrated effort on the procedural and documentary aspects of review conduct, while the interpretive, judgment-dependent step of weighing bias against the pooled estimate has received less attention. This is offered as a hypothesis, not a demonstrated mechanism: The two eras were rated by different teams, this domain had the lowest inter-rater agreement in our sample, and a between-era difference in rating thresholds cannot be excluded. If the pattern is real, it warrants attention from reviewers, editors, and appraisal-tool developers, because it is precisely the interpretive domains that protect readers from overconfident conclusions.

Two observations bear on how robust these findings are. First, the finding that most reviews remain critically low is robust to scoring convention: under the stricter rule, 25 of 26 contemporary reviews are critically low, compared with 16 of 26 under the primary rule and 79 of 81 in 2009-2019. The finding that a minority now reach low confidence or better is, by contrast, sensitive to that convention, falling from 10 of 26 to one of 26 under the stricter rule; we present both so readers can judge which claim they find most relevant. The item 13 difference is unaffected by this convention, as the item is scored identically under both rules. Second, the two principal findings, protocol reporting and risk of bias in interpretation, are the only between-era differences whose confidence intervals exclude zero by a wide margin. The smaller differences on other items, including the apparent fall in reporting the funding of included studies, have intervals spanning zero and should not be read as evidence of change. Excluding the dominant journal preserved the direction of the principal findings.

These findings sit alongside comparable AMSTAR-2 appraisals in other specialties, which have likewise found most systematic reviews to be of low or critically low confidence, and extend that literature by setting a contemporary sample beside an earlier one drawn from the same journal frame [31]. The persistence of a critically low rating for most reviews, even amid clear procedural improvement, is consistent with AMSTAR-2's demanding threshold, in which a single unmet critical domain caps the overall rating [1].

Limitations

This survey has limitations. The contemporary sample (n=26) is smaller than the comparator (n=81) and spans five years rather than 11. The reduction reflects both the shorter window and the stricter, population-based definition of heart failure applied here, and it raises the possibility that the contemporary sample is a more homogeneous subset of the literature than the earlier one; if reviews with less rigorous population definitions were disproportionately excluded, the comparison could be biased toward showing higher compliance. The pre-registered sensitivity analysis designed to test this was not performed, for the reasons given in Results, and this is disclosed as a protocol deviation. Two reviews were excluded from the initially frozen sample after peer review on eligibility grounds; both had been fully appraised before exclusion, their ratings are retained in the deposited dataset, and their removal did not change the direction of any principal finding.

The comparison is against the published aggregate figures of the earlier study rather than a re-rating of its sample, so a portion of any between-era difference could reflect differences between rating teams rather than a true change in practice. The two eras share the journal frame but differ in retrieval method, population definition, and appraisal team and are not a matched comparison; observed differences are reported as such and not attributed to time or to any intervention. Sixteen of 26 reviews originated from a single journal, although a sensitivity analysis excluding it preserved the direction of the principal findings. Inter-rater agreement was moderate overall and lowest on the interpretive domain underlying the principal negative finding, and its consensus value should be read with that subjectivity in mind. The scoring rule applied to AMSTAR-2 items 12 and 13 involves interpretive judgment and is stated in Materials & Methods so that ratings can be recomputed under an alternative reading. Finally, ratings could not be blinded to the publication year.

## Conclusions

AMSTAR-2 compliance of high-impact heart failure systematic reviews was higher in 2021-2025 than in a comparable 2009-2019 sample, most markedly for a priori protocol reporting, and a minority of contemporary reviews reached low confidence or better. Most reviews nonetheless remained critically low; the funding of individual included studies was rarely reported in either era, and contemporary reviews were less likely to account for risk of bias when interpreting their results. Whether this reflects a shift of effort from substantive appraisal toward procedural compliance cannot be established by this design, but the pattern suggests that authors, peer reviewers, and editors of heart failure evidence syntheses should attend not only to registration and reporting checklists but also to the interpretive judgments that those instruments are meant to safeguard.

## Appendices

Included studies

Table 2 shows the 28 heart failure systematic reviews with meta-analyses appraised in this survey, published from 2021 to 2025 in 11 high-impact journals [5-30]. 

**Table 2:** The 26 heart failure systematic reviews with meta-analysis appraised in this survey References [5-30], published 2021-2025 in 11 high-impact journals. Two records originally included (Bhatia et al. and Khan et al.) were excluded following a post-hoc eligibility audit prompted by peer review: the former synthesised trials across a mixed population of type 2 diabetes, chronic kidney disease and heart failure rather than a heart-failure-only population; the latter is an umbrella review re-pooling existing meta-analyses rather than a synthesis of primary studies. The complete author list is given for reviews with seven or fewer authors; for reviews with more than seven authors, the first three are listed followed by "et al." The complete item-level AMSTAR-2 ratings, including those of the two excluded reviews, and the analysis code are available on the Open Science Framework (https://doi.org/10.17605/OSF.IO/KNGQ5).

ID	Ref	Authors	Journal	Title	DOI
S1	[5]	Lauder L, Pereira TV, Degenhardt MC, et al.	Eur J Heart Fail	Feasibility and efficacy of transcatheter interatrial shunt devices for chronic heart failure: a systematic review and meta-analysis	10.1002/ejhf.2360
S2	[6]	Turgeon RD, Barry AR, Hawkins NM, Ellis UM	Eur J Heart Fail	Pharmacotherapy for HFrEF and health-related quality of life: a systematic review and meta-analysis	10.1002/ejhf.2141
S3	[7]	Vaduganathan M, Docherty KF, Claggett BL, et al.	Lancet	SGLT2 inhibitors in patients with heart failure: a comprehensive meta-analysis of five randomised controlled trials	10.1016/S0140-6736(22)01429-5
S4	[8]	Vukadinović D, Abdin A, Anker SD, et al.	Eur J Heart Fail	Side effects and treatment initiation barriers of SGLT2 inhibitors in heart failure: a systematic review and meta-analysis	10.1002/ejhf.2584
S5	[9]	Zito A, Princi G, Romiti GF, et al.	Eur J Heart Fail	Device-based remote monitoring strategies for congestion-guided management of patients with heart failure: a systematic review and meta-analysis	10.1002/ejhf.2655
S6	[10]	Zou X, Shi Q, Vandvik PO, et al.	Ann Intern Med	Sodium-glucose cotransporter-2 inhibitors in patients with heart failure: a systematic review and meta-analysis	10.7326/M21-4284
S7	[11]	Banerjee M, Maisnam I, Pal R, Mukhopadhyay S	Eur Heart J	Mineralocorticoid receptor antagonists with SGLT2 inhibitors in heart failure: a meta-analysis	10.1093/eurheartj/ehad522
S8	[12]	Clephas PRD, Radhoe SP, Boersma E, et al.	Eur Heart J	Efficacy of pulmonary artery pressure monitoring in patients with chronic heart failure: a meta-analysis of three randomized controlled trials	10.1093/eurheartj/ehad346
S9	[13]	Colin-Ramirez E, Sepehrvand N, Rathwell S, et al.	Circ Heart Fail	Sodium restriction in patients with heart failure: a systematic review and meta-analysis of randomized clinical trials	10.1161/CIRCHEARTFAILURE.122.009879
S10	[14]	Graham FJ, Pellicori P, Kalra PR, Ford I, Bruzzese D, Cleland JGF	Eur J Heart Fail	Intravenous iron in patients with heart failure and iron deficiency: an updated meta-analysis	10.1002/ejhf.2810
S11	[15]	Iaconelli A, Pellicori P, Dolce P, et al.	Eur J Heart Fail	Coronary revascularization for heart failure with coronary artery disease: a systematic review and meta-analysis of randomized trials	10.1002/ejhf.2911
S12	[16]	Modin D, Lassen MCH, Claggett B, et al.	Eur J Heart Fail	Influenza vaccination and cardiovascular events in patients with ischaemic heart disease and heart failure: a meta-analysis	10.1002/ejhf.2945
S13	[17]	Molloy CD, Long L, Mordi IR, et al.	Eur J Heart Fail	Exercise-based cardiac rehabilitation for adults with heart failure: 2023 Cochrane systematic review and meta-analysis	10.1002/ejhf.3046
S14	[18]	Pandey AK, Dhingra NK, Pandey A, et al.	Eur J Heart Fail	Efficacy of SGLT2 inhibitors and ARNIs for heart failure in Black patients: a systematic review and meta-analysis of randomized controlled trials	10.1002/ejhf.2825
S15	[19]	Scholte NTB, Gürgöze MT, Aydin D, et al.	Eur Heart J	Telemonitoring for heart failure: a meta-analysis	10.1093/eurheartj/ehad280
S16	[20]	Usman MS, Siddiqi TJ, Anker SD, et al.	J Am Coll Cardiol	Effect of SGLT2 inhibitors on cardiovascular outcomes across various patient populations	10.1016/j.jacc.2023.04.034
S17	[21]	Li J, Chen Y, Wang Y, et al.	Eur J Heart Fail	Impact of guideline-directed medical therapy on systolic blood pressure and cardiovascular outcomes in heart failure with low blood pressure: a systematic review and meta-analysis	10.1002/ejhf.3208
S18	[22]	Martens P, Nunes Augusto S Jr, Mullens W, Tang WHW	JACC Heart Fail	Meta-analysis and metaregression of the treatment effect of intravenous iron in iron-deficient heart failure	10.1016/j.jchf.2023.11.006
S19	[23]	Oraii A, Healey JS, Kowalik K, et al.	Eur Heart J	Mineralocorticoid receptor antagonists and atrial fibrillation: a meta-analysis of clinical trials	10.1093/eurheartj/ehad811
S20	[24]	Usman MS, Siddiqi TJ, Anker SD, et al.	Eur J Heart Fail	Effect of SGLT2 inhibitors on left cardiac remodelling in HFrEF: systematic review and meta-analysis	10.1002/ejhf.3129
S21	[25]	De Lathauwer ILJ, Nieuwenhuys WW, Hafkamp F, Regis M, Brouwers RWM, Funk M, Kemps HMC	Eur J Heart Fail	Remote patient monitoring in heart failure: a comprehensive meta-analysis of effective programme components for hospitalization and mortality reduction	10.1002/ejhf.3568
S22	[26]	Fioretti F, Praderio A, Nodari S, Metra M, Butler J	Eur J Heart Fail	Combination diuretic therapy in acute heart failure: a systematic review and meta-analysis	10.1002/ejhf.70094
S23	[27]	Kapelios CJ, Liori S, Bonios M, Abraham WT, Filippatos G	Eur J Heart Fail	Effect of pulmonary artery pressure-guided management on outcomes of patients with heart failure outside clinical trials: a systematic review and meta-analysis of real-world evidence (CardioMEMS)	10.1002/ejhf.3687
S24	[28]	Neves JS, Vale C, Leite AR, et al.	JACC Heart Fail	GLP-1 receptor agonists in heart failure with reduced ejection fraction: meta-analysis of randomized clinical trials	10.1016/j.jchf.2025.03.035
S25	[29]	Nguyen DV, Le TN, Truong BQ, Nguyen HTT	Eur J Heart Fail	Efficacy and safety of ARNI in heart failure patients with end-stage kidney disease on maintenance dialysis: a systematic review and meta-analysis	10.1002/ejhf.3454
S26	[30]	Ravikulan R, Chavali S, Gunton JE, De Pasquale CG	Eur J Heart Fail	GLP-1 receptor agonists reduce atrial fibrillation among patients with HFpEF and HFmrEF: a meta-analysis of randomized controlled trials	10.1002/ejhf.70085
